# *Clostridioides difficile* infection in the Asia-Pacific region

**DOI:** 10.1080/22221751.2019.1702480

**Published:** 2019-12-24

**Authors:** Deirdre A. Collins, Kyung Mok Sohn, Yuan Wu, Kentaro Ouchi, Yoshikazu Ishii, Briony Elliott, Thomas V. Riley, Kazuhiro Tateda, for the Clostridioides difficile Asia-Pacific Study Group

**Affiliations:** a School of Biomedical Sciences, The University of Western Australia, Perth, Australia; b Division of Infectious Diseases, Department of Internal Medicine, Chungnam National University School of Medicine, Daejeon, Republic of Korea; c State Key Laboratory for Infectious Disease Prevention and Control, Collaborative Innovation Center for Diagnosis and Treatment of Infectious Diseases, National Institute for Communicable Disease Control and Prevention, Chinese Center for Disease Control and Prevention, Beijing, People’s Republic of China; d Medical Affairs, Otsuka Pharmaceutical Co., Ltd, Osaka, Japan; e Department of Microbiology and Infectious Diseases, Toho University School of Medicine, Tokyo, Japan; f Department of Infection Control, Toho University Medical Center, Omori Hospital, Tokyo, Japan; g Department of Microbiology, PathWest Laboratory Medicine (WA), Perth, Australia; h Laboratory Microbiological Section, Toho University Medical Center, Omori Hospital, Tokyo, Japan

**Keywords:** *Clostridioides difficile* infection, Asia-Pacific, epidemiology, clinical features, molecular epidemiology

## Abstract

*Clostridioides difficile* causes healthcare-related diarrhoea in high-income countries. Highly resistant spores persist in healthcare facilities, primarily infecting patients who have recently received antimicrobials. *C. difficile* infection (CDI) has been studied in detail in North America and Europe; however, the epidemiology of CDI elsewhere, including the Asia-Pacific region, is largely unknown. A survey of CDI was performed in 13 Asia-Pacific countries. Epidemiological data on 600 cases were collected and molecular typing undertaken on 414 *C. difficile* isolates. Healthcare facility-associated CDI comprised 53.6% of cases, while community-associated CDI was 16.5%. The median age of cases was 63.0 years and 45.3% were female, 77.5% had used antibiotics in the previous 8 weeks, most frequently third-generation cephalosporins (31.7%), and 47.3% had used proton pump inhibitors. Recurrence (9.1%) and mortality (5.2%) rates were low, while complications including colitis or pseudomembranous colitis (13.8%), colectomy (0.4%), and toxic megacolon (0.2%) were uncommon. Common *C. difficile* strains were ribotypes 017 (16.7%), 014/020 (11.1%) and 018 (9.9%), with wide variation between countries. Binary toxin-positive strains of *C. difficile* were detected rarely. Overall, disease severity appeared mild, and mortality and recurrence were low. Continued education about, and surveillance of, CDI in Asia are required to reduce the burden of disease.

## Introduction

*Clostridioides* (also called *Clostridium*) *difficile* [1] is a major cause of healthcare-related infection in high-income countries [2]. *C. difficile* infection (CDI) can manifest in a wide range of symptoms from mild diarrhoea to life-threatening pseudomembranous colitis (PMC) and toxic megacolon. The disease is mediated by the production of toxins with most strains of *C. difficile* producing two toxins, toxins A (TcdA/enterotoxin) and B (TcdB/cytotoxin) [3]. Some *C. difficile* strains cause disease while producing only one toxin, toxin B [4], while a small but increasing proportion of strains produce a third toxin (binary toxin, CDT), the importance of which is still unclear [5]. The primary risk factors for CDI have been antimicrobial exposure, hospital admission, and advanced age. Major epidemics of CDI have been recorded in Europe and North America over recent decades following the emergence of a fluoroquinolone-resistant, binary toxin-producing (CDT+) strain of *C. difficile* (ribotype/RT 027, multilocus sequence type [MLST] ST 1) [3]. Thus, most epidemiological studies on CDI to date have been reported from these regions, with a minority of reports from Latin and South America, and Africa.

Limited knowledge exists of the epidemiology of CDI in Asia-Pacific countries, due to a general lack of awareness among physicians and inappropriate testing in some places [6]. Given recent economic growth in many Asian countries, with ageing populations, increased access to healthcare and widespread inappropriate use of antimicrobials [7], *C. difficile* is likely to be a significant cause of disease in the region. International epidemics of CDI have highlighted the need for monitoring the spread of *C. difficile*. While the highly publicized outbreaks in North America and Europe were caused by a clonal strain of RT 027 *C. difficile* originating in North America [8], significant outbreaks of RT 017 (ST 37) *C. difficile* [9–11], the most common strain in Asia, demonstrated the potential for international spread of *C. difficile* strains to and from the region. Interestingly, *C. difficile* RT 027 has rarely been reported from Asia [6,12].

The *C. difficile* population structure consists of the phylogenetic clades 1 through 5 and clades C-I, C-II, and C-III [13,14]. Clades 1–5 appear to associate with particular geographical regions of the world (e.g. clade 2 with North America). Clade 4 is dominated by largely non-toxigenic strains, except for RT 017 which only produces toxin B (A−B+) [13]. *C. difficile* RT 017 has been the predominant strain identified in most Asian studies to date, particularly in Northern Asia and China [4,6]. Recent studies in Malaysia [15], Indonesia [16], and Thailand [17] have shown a high prevalence of RT 017 together with many non-toxigenic strains in Southeast Asia, supporting the importance of Clade 4 strains in the region. Another A−B+ strain, RT 369, has also been reported in significant numbers from Japan [18,19]. Binary toxin-producing strains of *C. difficile* in general have been recorded rarely in Asia, further suggesting a regional predominance of clade 4 strains [6].

Laboratory testing for CDI has been infrequent in some Asian countries. In the past, many Asian studies reported using enzyme immunoassay (EIA) tests for TcdA to detect *C. difficile*. The abundance of A−B+ strains in the region meant that much of the testing that was undertaken in Asia was inappropriate and most likely led to under-diagnosis of CDI, and a misconception that *C. difficile* was perhaps an insignificant cause of diarrhoea in the region [20]. Studies from Singapore have illustrated how an apparent increase in the incidence of CDI (ranging from 1.49 cases per 10,000 patient days [PD] in 2001 to 10.7/10,000 PD in 2012) was partly attributable to increased awareness, increased testing and the introduction of more sensitive diagnostic methods such as polymerase chain reaction (PCR) for *tcdB* [21,22].

We conducted a multi-country, prospective observational study to characterize patients with CDI across the Asia-Pacific region as well as recording treatment and management of the disease. We also aimed to describe the molecular epidemiology of *C. difficile* strains isolated in the Asia-Pacific region.

## Materials and methods

### Study setting

The descriptive study was conducted at 40 hospital sites across 13 countries: Australia, China, Hong Kong, India, Indonesia, Japan, Malaysia, the Philippines, Singapore, South Korea, Taiwan, Thailand, and Vietnam. Cases were recruited from March 2014 to January 2015 with a target of 100 cases per country.

### Data collection

A CDI case was defined as a patient with at least three episodes of diarrhoea (unformed stool assuming its container's shape) within a 24 h period, with detection of free toxin in the stool by EIA or cell culture cytotoxicity assay (CCCA), detection of toxigenic *C. difficile* by direct real-time-PCR (RT-PCR) for *tcdB* or toxigenic culture, or colonoscopic findings of PMC [23]. Patients with diarrhoea caused by bacteria other than *C. difficile* were excluded. Cases were identified according to the above criteria, then invited to participate in the study, and then they or their legal guardian provided informed consent for inclusion. Clinical laboratory data and information on testing methodology, patient demographics, medical history, medications, and disease characteristics and outcomes were collected by medical record review and a follow-up phone call or record review 2 months after treatment. Approval to perform the study was granted by the relevant Human Research Ethics Committee at each participating site.

Healthcare-associated cases (HA-CDI) were defined as CDI occurring >48 h after admission or within 4 weeks of discharge. Community-associated cases (CA-CDI) occurred in the community or within the first 48 h of hospital admission, with no history of hospital admission within the past 12 weeks. Cases were defined as “indeterminate” where hospitalization had occurred in the previous 4–12 weeks. Recurrent CDI was defined as a CDI episode occurring within 8 weeks of a previous, resolved episode [23]. Severe disease was defined by a white cell count (WCC) ≥15,000 cells/µL or peak serum creatinine level 1.5 times greater than the premorbid level or, if not recorded, 1.5 times greater than the normal range. Complicated severe disease was defined as hypotension, shock, ileus, or megacolon [23].

### *C. difficile* culture and molecular analysis

Following recruitment to the study, stool samples were collected for cases and transported on swabs in Cary Blair medium at ambient temperature to a central processing laboratory (LSI Medience, Tokyo, Japan), or local reference laboratories in Australia and China (PathWest Laboratory Medicine WA, Perth, Australia; Chinese Center for Disease Control and Disease Prevention, Beijing, China). National restrictions prevented samples from India from being transported to a processing laboratory for the study. Samples were tested for free toxin by EIA (C. diff Chek Complete, TechLab, Inc., Blacksburg, VA) and cultured directly on ChromID™ *C. difficile* agar (bioMérieux, France) and incubated anaerobically at 35°C for 48 h. Samples were also enriched in cycloserine-cefoxitin fructose broth supplemented with 0.1% sodium taurocholate and incubated for 7 days, followed by subculture on ChromID™ *C. difficile* agar. Putative *C. difficile* colonies were confirmed by Rapid ID 32A (bioMérieux, Marcy-l’Etoile, France) or morphology, odour, and chartreuse fluorescence on blood agar.

DNA was extracted from pure cultures on blood agar plates, and PCR ribotyping [24] and detection of the toxin genes *tcdA*, *tcdB*, *cdtA*, and *cdtB* [25,26] were performed. PCR ribotyping products were resolved on the QIAxcel capillary electrophoresis platform (QIAGEN, Venlo, Limburg, Netherlands). The resulting band profiles were compared with a reference collection by cluster analysis using BioNumerics™ v.7.6 (Applied Maths, Sint-Martens-Latem, Belgium).

### Data analysis

Data were recorded in a centrally monitored online data capture form. All descriptive and statistical analyses were performed using SPSS v22.0 (IBM Corp., Armonk, NY, USA). Cases <2-year-old, and cases where an A−B− isolate or no isolate was recovered, were excluded from risk factor analyses. Univariate odds ratios (ORs) were calculated for factors that could be associated with outcomes of severe or recurrent CDI. Including variables with univariate OR with *p *< .2, multivariable analyses were performed by logistic regression using a generalized linear mixed model accounting for country and site as random effects, given that patient characteristics were likely to vary between sites and countries.

## Results

### Patient demographics

In total, 600 patients were recruited to the study across Australia, China, Hong Kong, India, Indonesia, Japan, Malaysia, the Philippines, Singapore, South Korea, Taiwan, Thailand, and Vietnam (Table 1). The target of 100 patients per country was not reached because of late commencement of recruitment in some regions due to delays in receiving local ethical approvals to conduct the study. Patients ranged in age from <1 to 105 years, with a median of 63.0 years (interquartile range [IQR] 45.0–75.0). Females accounted for 45.3% of participants overall (Table 2).

**Table 1.** Number of participating sites and recruited patients per country.

Country	Participating sites (*n*)	Total recruited participants *n* (%)
Australia	3	60 (10.0)
China	8	64 (10.7)
Hong Kong	3	36 (6.0)
India	1	2 (0.3)
Indonesia	1	7 (1.2)
Japan	3	48 (8.0)
Malaysia	2	2 (0.3)
Philippines	3	9 (1.5)
Singapore	3	66 (11.0)
South Korea	3	99 (16.5)
Taiwan	5	99 (16.5)
Thailand	3	44 (7.3)
Vietnam	2	64 (10.7)
Total	40	600 (100.0)

**Table 2.** Patient demographics and CDI characteristics.

Characteristic	*n* (%)
HA-CDI	359 (53.6)
CA-CDI	96 (16.5)
Indeterminate	128 (22.0)
Recurrent CDI	36 (6.0)
Median length of stay (days) [IQR]	22.0 [10.0–43.0]
Female	272 (45.3)
Age range	
0–14	75 (12.5)
15–29	24 (4.0)
30–49	71 (11.8)
50−64	150 (25.0)
65–79	185 (30.8)
80+	95 (15.8)
Disease characteristics	
Toxin A/B detected	224 (78.0)
Blood in stool	93 (15.5)
Abdominal pain	191 (31.8)
Fever (temperature ≥38.5°C)	34 (5.9)
Peak white cell count ≥15,000 cells/µL	128 (26.8)
Peak serum creatinine ≥1.5 times normal range	104 (22.6)
Severe CDI	191 (31.8)
Complicated severe CDI	9 (1.7)
Medical history	
Myocardial infarction	54 (9.0)
Congestive heart failure	46 (7.7)
Cerebrovascular disease	63 (10.5)
Peripheral vascular disease	29 (4.8)
Chronic pulmonary disease	57 (9.5)
Connective tissue disease	72 (12.0)
Peptic ulcer disease	69 (11.5)
Liver disease	76 (12.7)
Diabetes mellitus	154 (25.7)
Renal disease	143 (23.8)
Solid tumour	145 (24.2)
Leukaemia	44 (7.3)
Lymphoma	39 (6.5)
AIDS	3 (0.5)
IBD	68 (11.3)
Nasogastric tube in past 30 days	107 (17.8)
Abdominal surgery in past 30 days	36 (6.0)
Medication in previous 8 weeks	
Chemotherapy	91 (15.2)
Proton pump inhibitor	284 (47.3)
Probiotic	115 (19.2)
NSAID	116 (19.3)
Steroid	196 (32.7)
Immune modulator	55 (9.2)
H_2_ receptor agonist	102 (17.0)
Antibiotic use in previous 8 weeks	465 (77.5)
Amoxicillin/amoxicillin clavulanate	103 (17.2)
First-generation cephalosporin	40 (6.7)
Second-generation cephalosporin	56 (9.3)
Third-generation cephalosporin	190 (31.7)
Fourth-generation cephalosporin	55 (9.2)
Fluoroquinolones	145 (24.2)
Trimethoprim/sulfamethoxazole	44 (7.3)
Metronidazole	46 (7.7)
Piperacillin/tazobactam	119 (19.8)
Carbapenems	120 (20.0)
Clindamycin	11 (1.8)
Vancomycin	73 (12.2)
Complications	
Emergency colectomy performed	2 (0.4)
Colonoscopy/sigmoidoscopy performed	2 (0.4)
Colitis or PMC	83 (13.8)
Toxic megacolon	1 (0.2)
Hypokalaemia	23 (4.4)
Hypotension	1 (0.2)
Dehydration	17 (3.2)
Septic shock	9 (1.7)
Peritonitis	4 (0.8)
Treatment	
Severe CDI	
Metronidazole	182 (97.3)
Vancomycin	88 (47.1)
Metronidazole and vancomycin	53 (29.9)
Non-severe CDI	
Metronidazole	224 (92.6)
Vancomycin	71 (29.3)
Metronidazole and vancomycin	36 (14.9)
Severity unknown	
Metronidazole	95 (91.3)
Vancomycin	20 (19.2)
Metronidazole and vancomycin	7 (6.7)
Outcomes	
Recurrent CDI	48 (9.1)
Death	31 (5.2)
Lost to follow-up	73 (12.2)

Note: Denominators vary due to missing data.

### Testing methods

RT-PCR was the most common diagnostic method (41.2%), most frequently used in Australia (100.0%), Taiwan (80.8%), Hong Kong (77.8%), and Singapore (74.2%). Toxin EIA (31.2% overall) was used most frequently in Vietnam (100.0%), India (100.0%), Indonesia (85.7%), the Philippines (66.7%), and Japan (62.5%). Toxigenic culture (26.0% overall) was most frequent in South Korea (89.9%) and China (60.9%). The remaining cases (1.7%) were detected by CCCA or colonoscopy.

### Disease characteristics

The characteristics of CDI patients in the study are described in Table 2. The majority of cases were HA-CDI (53.6%), while 16.5% were CA-CDI, 22.0% were indeterminate, while the remaining 7.9% could not be classified due to missing data. Recurrent CDI was identified in 36 cases (6.0%). The median length of hospital stay was 22.0 days (IQR 10.0–43.0). Abdominal pain was experienced in 31.8% of participants, while blood was reported in the stool of 15.5%. Markers of severe CDI were recorded in 191 cases (31.8%), most frequently indicated by elevated WCC (26.8%), then elevated serum creatinine level (22.6%). Complicated severe infection was identified in nine cases (1.7%) (Table 2).

### Medical history

The most common comorbidities were diabetes mellitus (25.7%), solid tumour (24.2%), and renal disease (23.8%). Over the previous month, nasogastric feeding had occurred in 107 cases (17.8%), while 36 (6.0%) underwent abdominal surgery. In the previous 8 weeks, 284 cases (47.3%) used proton pump inhibitors (PPIs), 196 (32.7%) steroids, 102 (17.0%) H_2_ receptor agonists, 116 (19.3%) non-steroidal anti-inflammatory drugs (NSAIDs), 115 (19.2%) probiotics, and 55 (9.2%) an immune modulator. Chemotherapy was administered to 91 cases (15.2%), while antibiotic use was recorded in 465 (77.5%), most commonly third-generation cephalosporins (31.7%), fluoroquinolones (24.2%), carbapenems (20.0%), piperacillin/tazobactam (19.8%), or amoxicillin/clavulanate (17.2%) (Table 2).

### Outcomes of CDI

Disease resulted in colectomy in two cases (0.4%), while toxic megacolon was recorded in one (0.2%). Colitis or PMC was recorded in 83 cases (13.8%). After the 2-month follow-up, recurrent CDI was recorded in 48 cases (9.1%), 42 of which had resolved and two of which had died at follow-up. Death within 2 months occurred in 31 cases (5.2%) overall, while loss to follow-up occurred for 73 patients (12.2%) (Table 2).

### Risk factor analysis

Severe CDI was univariately associated with history of myocardial infarction (OR 3.41, 95% CI 1.59–7.31), diabetes mellitus (2.29, 95% CI 1.40–3.75), and renal disease (6.60, 95% CI 3.80–11.45), use of PPIs (1.83, 95% CI 1.16–2.89), third-generation cephalosporins (1.83, 95% CI 1.13–2.95), or vancomycin (3.64, 95% CI 1.75–7.55; Table 3). By multivariable analysis, only myocardial infarction (8.78, *p *< .01) was significantly associated with severe CDI.

**Table 3.** Risk of the outcome of severe CDI.

	Univariate OR (95% CI)	*p*	Multivariable analysis OR	*p*
HA-CDI	0.69 (0.42–1.14)	.15	2.33	.13
CA-CDI	0.96 (0.44–2.13)	.93		
Recurrent CDI (CDI episode in previous 8 weeks)	1.38 (0.54–3.48)	.50		
Age ≥65 years	1.38 (0.88–2.18)	.16	1.34	.25
Sex	0.95 (0.61–1.50)	.84		
Disease characteristics				
Free toxin detected	0.56 (0.30–1.03)	.06	1.59	.21
Blood in stool	1.27 (0.69–1.34)	.45		
Abdominal pain	1.22 (0.76–1.96)	.42		
*C. difficile* strain				
RT 017	1.53 (0.82–2.85)	.18	1.30	.26
RT 014/020	0.97 (0.49–1.95)	.94		
RT 018	0.63 (0.30–1.32)	.22		
RT 002	1.21 (0.59–2.47)	.61		
RT 012	0.81 (0.29–2.24)	.68		
RT 369	0.44 (0.14–1.39)	.16	0.07	.80
QX 239	0.24 (0.05–1.08)	.06	2.70	.10
QX 032	2.49 (0.80–7.78)	.12	1.01	.32
Medical history				
Myocardial infarction	3.41 (1.59–7.31)	.002	8.78	.003
Congestive heart failure	2.29 (1.06–4.98)	.04	2.16	.14
Peripheral vascular disease	2.73 (1.04–7.13)	.04	1.69	.20
Cerebrovascular disease	1.16 (0.54–2.48)	.70		
Chronic pulmonary disease	1.68 (0.82–3.46)	.16	1.15	.29
Connective tissue disease	1.07 (0.55–2.08)	.85		
Peptic ulcer disease	1.49 (0.75–2.96)	.26		
Liver disease	0.94 (0.50–1.75)	.84		
Diabetes mellitus	2.29 (1.40–3.75)	.001	2.45	.12
Renal disease	6.60 (3.80–11.45)	<.001		
Solid tumour	1.36 (0.83–2.24)	.23		
Leukaemia	0.69 (0.30–1.57)	.37		
Lymphoma	0.77 (0.35–1.71)	.52		
AIDS	1.50 (0.09–24.20)	.78		
IBD	0.83 (0.40–1.70)	.61		
Nasogastric tube in past 30 days	2.22 (1.24–3.97)	.007	0.11	.74
Abdominal surgery in past 30 days	1.42 (0.63–3.22)	.40		
Medication in previous 8 weeks				
Chemotherapy	0			
Proton pump inhibitor	1.83 (1.16–2.89)	.009	1.06	.31
Probiotic	0.71 (0.38–1.31)	.27		
NSAID	0			
Steroid	0			
Immune modulator	0			
H_2_ receptor agonist	1.35 (0.89–2.30)	.28		
Antibiotic use in previous 8 weeks	1.36 (0.90–2.32)	.26		
Amoxicillin/amoxicillin clavulanate	1.29 (0.68–2.46)	.43		
First-generation cephalosporin	0.78 (0.34–1.80)	.56		
Second-generation cephalosporin	0.69 (0.30–1.57)	.37		
Third-generation cephalosporin	1.83 (1.13–2.95)	.01	0.03	.87
Fourth-generation cephalosporin	1.30 (0.64–2.63)	.47		
Fluoroquinolones	1.03 (0.59–1.80)	.92		
Trimethoprim/sulfamethoxazole	0.73 (0.33–1.61)	.43		
Metronidazole	1.91 (0.77–4.74)	.17	0.33	.57
Piperacillin/tazobactam	1.62 (0.95–2.77)	.08	0.01	.93
Carbapenems	1.46 (0.86–2.48)	.16	0.45	.50
Clindamycin	0.37 (0.04–3.34)	.38		
Vancomycin	3.64 (1.75–7.55)	.001	2.78	.10

Outcome of recurrent CDI was associated with QX 239 (3.92, 95% CI 1.15–13.41), QX 032 (5.05, 95% CI 1.60–15.93), a previous recurrent CDI episode (2.71, 95% CI 1.02–7.22), fluoroquinolone (2.22, 95% CI 1.09–4.52), trimethoprim/sulfamethoxazole (3.16, 95% CI 1.31–7.63), and carbapenem (2.69, 95% CI 1.33–5.43) use (Table 3). Only QX 239 (5.04, *p *< .01) was significantly associated with an outcome of recurrence by multivariable analysis (Table 4).

**Table 4.** Risk of the outcome of recurrent CDI at 2-month follow-up.

	Univariate OR (95% CI)	*p*	Multivariable analysis OR	*p*
HA-CDI	0.92 (0.45–1.90)	.83		
CA-CDI	0.73 (0.21–2.51)	.62		
Recurrent CDI (CDI episode in previous 8 weeks)	2.71 (1.02–7.22)	.05	1.68	.20
Age ≥65 years	1.21 (0.61–2.37)	.59		
Sex	0.94 (0.48–1.83)	.86		
Disease characteristics				
Free toxin detected	2.74 (0.80–9.35)	.11	3.20	.08
Severe CDI	1.19 (0.58–2.47)	.64		
*C. difficile* strain				
RT 017	0.89 (0.36–2.23)	.80		
RT 014/020	1.61 (0.67–3.91)	.29		
RT 018	1.19 (0.44–3.24)	.74		
RT 002	0.45 (0.10–1.96)	.29		
RT 012	0.44 (0.06–3.38)	.43		
RT 369	2.13 (0.57–7.89)	.26		
QX 239	3.92 (1.15–13.41)	.03	5.04	.03
QX 032	5.05 (1.60–15.93)	.01	3.67	.06
Medical history				
Myocardial infarction	1.10 (0.67–2.30)	.87		
Congestive heart failure	0.54 (0.12–2.35)	.41		
Peripheral vascular disease	0.44 (0.06–3.38)	.43		
Cerebrovascular disease	1.47 (0.58–3.76)	.42		
Chronic pulmonary disease	1.36 (0.50–3.74)	.55		
Connective tissue disease	0.61 (0.18–2.09)	.44		
Peptic ulcer disease	0.82 (0.28–2.43)	.72		
Liver disease	2.03 (0.90–4.57)	.09	0.37	.55
Diabetes mellitus	0.50 (0.21–1.17)	.11	1.12	.29
Renal disease	0.88 (0.40–1.93)	.75		
Solid tumour	1.27 (0.62–2.57)	.51		
Leukaemia	1.89 (0.67–5.28)	.23		
Lymphoma	1.65 (0.60–4.58)	.34		
AIDS	8.34 (0.51–136.11)	.14	0.59	.45
IBD	1.66 (0.68–4.03)	.26		
Nasogastric tube in past 30 days	0.87 (0.37–2.06)	.75		
Abdominal surgery in past 30 days	1.12 (0.32–3.93)	.86		
Treatment				
Metronidazole	1.91 (0.61–5.95)	.27		
Vancomycin	0			
Metronidazole and vancomycin	2.03 (0.90–4.57)	.09	2.83	.10
Probiotic	0			
FMT	0			
Medication in previous 8 weeks				
Chemotherapy	0			
Proton pump inhibitor	1.64 (0.84–3.21)	.15	0.42	.52
Probiotic	1.38 (0.60–3.18)	.45		
NSAID	0			
Steroid	0			
Immune modulator	0			
H_2_ receptor agonist	1.40 (0.65–3.01)	.40		
Antibiotic use in previous 8 weeks	0.94 (0.44–2.02)	.88		
Amoxicillin/amoxicillin clavulanate	0.89 (0.33–2.40)	.82		
First-generation cephalosporin	1.72 (0.62–4.80)	.30		
Second-generation cephalosporin	0.96 (0.32–2.85)	.93		
Third-generation cephalosporin	0.92 (0.45–1.88)	.81		
Fourth-generation cephalosporin	2.16 (0.92–5.07)	.08	0.32	.57
Fluoroquinolones	2.22 (1.09–4.52)	.03	1.49	.22
Trimethoprim/sulfamethoxazole	3.16 (1.31–7.63)	.01	1.65	.20
Metronidazole	1.67 (0.57–4.91)	.35		
Piperacillin/tazobactam	0.87 (0.38–1.97)	.73		
Carbapenems	2.69 (1.33–5.43)	.006	1.19	.28
Clindamycin	1.65 (0.19–14.48)	.65		
Vancomycin	0.99 (0.33–2.95)	.98		

### Treatment

Patients were most frequently treated with metronidazole (94.0%), followed by vancomycin (33.6%); some patients were treated with a combination of both, most frequently in severe cases (18.0% overall, Table 2). A minority of patients were treated with probiotics (0.7%), while three patients (two in Singapore, one in China) underwent faecal microbiota transplant (FMT) (0.5%).

### Culture and molecular epidemiology

**Figure 1.**
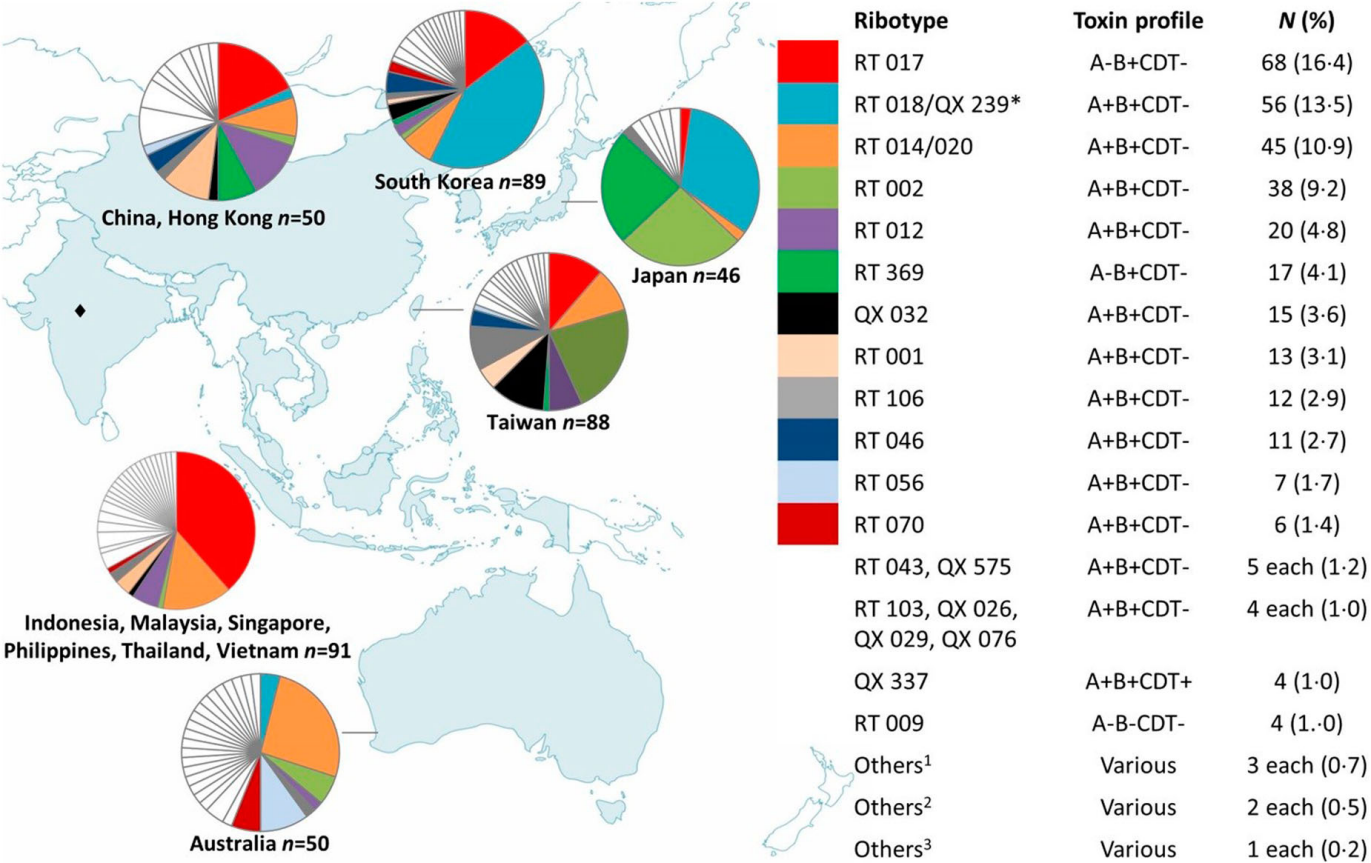
RT frequencies by country/region; 414 isolates were included. *n*: number of isolates. *RT 018 and QX 239 differed by one band. QX 239 corresponds to smz’ (H Kato, personal communication) and was only isolated in Japan, RT 018 was not isolated in Japan. Since recruitment numbers were low for South-East Asian countries and China and Hong Kong, data were pooled for these regions. ♦No isolates were collected for India. ^1^QX 020 (A+B+CDT−); RT 027, QX 449 (A+B+CDT+). ^2^RT 005, RT 015, RT 053, RT 131, RT 137, QX 237 (A+B+CDT−); RT 078, QX 404 (A+B+CDT+); QX 510 (A−B−CDT−). 3RT 049, QX 005, QX 033, QX 041, QX 051, QX 066, QX 068, QX 070, QX 153, QX 193, QX 366, QX 417, QX 439, QX 474, QX 497, QX 577, QX 583, QX 592 (A+B+CDT−); RT 127, QX 052, QX 055, QX 273, QX 408, QX 480, QX 587 (A+B+CDT+); QX 058, QX 473, QX 568, QX 590 (A−B+CDT−); RT 010, QX 011, QX 077, QX 095, QX 107, QX 427, QX 463, QX 471, QX 508, QX 567, QX 578, QX 579, QX 580, QX 581, QX 588, QX 591 (A−B−CDT−).

A total of 414 isolates were recovered by culture. Among 79 RTs, the most common were RTs 017 (16.4%), 018/QX 239 (13.5%; 9.9% and 3.6%, respectively), 014/020 (10.9%), and 002 (9.2%), while 46 RTs were represented by singleton strains (Figure 1). A−B+ strains (26.8%) were mostly RTs 017 and 369 (4.1%), 22 isolates (5.3%) were A−B−.

CDT+ strains of *C. difficile* were rare in the isolate collection with a total of only 22 isolates (5.3%; Figure 1). *C. difficile* QX 337 was isolated in Japan and Taiwan (one isolate each), and South Korea (two isolates), and QX 449 from South Korea (one isolate) and Thailand (two isolates). *C. difficile* RT 027 was only identified in the Philippines (two isolates from the same hospital) and China (one isolate). RT 127 was isolated once from Taiwan, while RT 078 was isolated once from Taiwan and once from South Korea; QX 404 was isolated twice from the same hospital in Taiwan. Other singleton CDT+ strains were isolated from Australia (QX 273 and QX 408), Japan (QX 033), South Korea (QX 052 and QX 480), and Taiwan (QX 587 and QX 055).

## Discussion

This is a large multi-national report on the descriptive epidemiology of CDI in Asia-Pacific countries. CDI cases in this study exhibited characteristics typical of CDI cases in other regions including advanced age, recent antimicrobial use, treatment with metronidazole and/or vancomycin, and a predominance of HA-CDI among cases.

It appears that while the reported prevalence of *C. difficile* in some Asia-Pacific countries is high [16,17,27], milder outcomes are associated with CDI in the region. In our study, toxic megacolon and colectomy were extremely rare (0.2% and 0.4%, respectively), while all-cause death occurred within 60 days in only 5.2% of cases, compared to 9.3% within 30 days in HA-CDI cases in the USA [28] and 22% within 90 days in a similar European study [29]. Recurrent infection occurred in 9.1% of cases, again less frequently than reported in Europe (16%) [29] and the USA (21%) [28].

The molecular epidemiology of strains isolated in this study was diverse and varied across the region (Figure 1). It also differed markedly to other regions where *C. difficile* is well-characterized, such as North America where RTs 027, 014/020, 002, 106, and 001 have predominated [28,30,31] and Europe, where RTs 027, 014, 001, and 078 have been isolated frequently [32]. Less is known of the molecular epidemiology of *C. difficile* in South America, but RTs 027, 106, 012, 046, and 014/020 are reportedly the most common strains [31,33,34]. Similar to the current study, RT 017 (followed by RT 001) has also been found at high prevalence in South Africa [35], and in Ghana, Malawi, and Tanzania the few studies which performed molecular typing have shown a high prevalence of non-toxigenic strains [36,37], similar to reports from Southeast Asian countries [15].

*C. difficile* RT 017 was the most common strain of *C. difficile* identified across the entire Asia-Pacific collection of isolates, and the most common A−B+ strain reported to date. Another A−B+ strain, RT 369, localized to northern Asia, particularly Japan (Figure 1). Coupled with many previous reports of RT 017 [6,17,38] and recent reports of RT 369 [18,19] in Asian countries, it appears that A−B+ strains are endemic to the region and a recent review of RT 017 concluded that the global spread of this MLST Clade 4 lineage member is a relatively recent event [4]. However, these findings contradict an earlier study of Cairns et al. [39] who used phylogeographic analyses to suggest a North American origin for RT 017.

*C. difficile* RT 018 also appears to be endemic to northern Asian countries (Figure 1), having predominated in South Korea since 2009 [6], and in Japan since the earliest typing studies, where it was referred to as “smz” [6,18]. A few RT 018 isolates were identified in Australia, where the molecular epidemiology differed in general from Asian countries. Strain QX 239, which was only isolated in Japan across three hospitals, differed in banding pattern from RT 018 by a single band and corresponds to smz’ (H Kato, personal communication), a strain which is closely related to RT 018 [18]. *C. difficile* QX 239 was significantly associated with disease recurrence (Table 4), which may have been due to enhanced antimicrobial resistance; however, susceptibility testing was not performed as part of this study. Greater antimicrobial resistance has been reported in RT 018 in Italy [40] and in a close relative, RT 356 [32], in Europe.

To date, reports of RT 027 in Asian countries have been infrequent [12]. The epidemic lineages of *C. difficile* RT 027 that caused large outbreaks after spreading from North America to Europe were fluoroquinolone-resistant, but RT 027 strains isolated in Asia have largely been fluoroquinolone-susceptible, caused sporadic disease only and may lack some other characteristics which enhanced the virulence of the epidemic RT 027 strain [8]. The rarity of RT 027 and CDT+ isolates in general in Asia suggests that cases may be imported sporadically from other regions of the world.

We also found several non-toxigenic strains (A−B−; 5.5%). These could have been false positives in diagnostic tests, or else were carried simultaneously with a toxigenic strain, then randomly selected during subculture of isolates. Due to uncertainty about whether they were carried with a toxigenic strain, cases, where they were isolated, were excluded from the logistic regression analyses. The prevalence of colonization with non-toxigenic strains in Asian countries appears to be high among hospital inpatients (10.4–28.6%) [15–17,27,41]. This is interesting given that clinical trials have found that administration of non-toxigenic strains reduces risk of recurrent infection in CDI cases [42], making it plausible that the high prevalence of colonization with non-toxigenic strains in Asia-Pacific countries may explain the low rate of recurrent CDI found in this study. In the present study, an outcome of recurrence did not occur in any case where a non-toxigenic strain was identified; however, as discussed above, it cannot be ascertained whether these were false positives or carried simultaneously with toxigenic strains. Elsewhere in the world, reports on non-toxigenic strains are rare, which may mean they are not as common in other regions, e.g. the prevalence of non-toxigenic strains was 2% in Australia [43]; however, this is more likely to be a result of publication bias towards toxigenic strains. Nonetheless, the high prevalence of non-toxigenic strains and A−B+ RTs 017 and 369 in Asia adds weight to the notion that Clade 4 strains are associated with the continent.

The molecular epidemiology of *C. difficile* strains identified in this study may contribute to the apparent milder outcomes of CDI in the region. A predominance of more virulent strains may be associated with more severe disease outcomes in North American and European countries, e.g. the prevalent RTs 027 and 078 [29,30]. In Europe, RTs 056 and 018 have caused severe or complicated infection [29,40,44]. In our analyses, no specific RT was associated with severe CDI (Table 3), nor with outcomes of complicated infection or death (data not shown).

A major limitation of this study is the fact that we were unable to calculate the incidence of CDI for participating sites and countries. In addition, some sites commenced recruitment several months later than others, due to delays in receiving approvals to conduct the study, resulting in poorer recruitment numbers in those countries, that included India, the Philippines, Indonesia, and Malaysia, resulting in incomplete coverage of the Asia-Pacific region which may confound some of our findings. A hierarchical structure was applied to our logistic regression models to account for variations between countries and sites to reduce risk of any confounding the skewed recruitment numbers may have caused. Since the diagnostic method used across countries varied, this could have introduced bias towards some patients who were colonized with *C. difficile* being recruited to the study. However, the case definition required ≥3 episodes of diarrhoea in 24 h, an unformed stool sample to be tested, and patients with laboratory findings of other bacterial diarrhoeal pathogens to be excluded from the study to minimize such a bias. Toxin A/B was detected in only 78% of cases, however (Table 2), which suggests that 22% may have been colonized rather than infected with *C. difficile*.

Another limitation was that cases with more severe outcomes may have been missed due to the requirement for informed consent in the study, as patients who were considered too ill to consent would not have been approached, and their guardians may have declined to consent to their recruitment. Otherwise, other population-specific factors may play a role in milder CDI outcomes and low recurrence rates, including diverse gut microbiota, coinfection with other agents which could be more common in the region, or region-specific differences in comorbidities or their management. Several (9) paediatric cases were recruited to the study in some countries; however, these were excluded from risk factor analyses. Another minor limitation is that the proportion of cases with pseudomembranous colitis could not be delineated from cases with colitis alone.

Notwithstanding these limitations, this study has many strengths. Prior to this investigation, few multi-country studies on CDI in the Asia-Pacific region existed. Multiple sites were included in most countries in the present study, giving a varied patient population for study. As a result, a large sample size was achieved, and standardized contemporaneous data collection and centralized data management across all countries involved ensured high-quality data were obtained. The study showed that it is vital to perform appropriate, consistent testing to identify patients with CDI in the region. CDI patients in Asia-Pacific countries exhibited typical characteristics of CDI as seen elsewhere; however, outcomes appeared to be less severe in Asia-Pacific countries. The molecular types of *C. difficile* causing disease in the region also differed markedly to North America and Europe. The A−B+ RTs of *C. difficile* 017 and 369 appear to be endemic to the region, and the previously demonstrated ability of RT 017 to acquire antimicrobial resistance and readily spread around the world [4] should be a cause for concern. Further investigations are required to determine the possible reasons for, and implications of, these findings, and continued surveillance will allow elucidation of international movement of strains, and identification of emerging types.

## CDAP Study Group

*Australia*: Michael Leung; PathWest Laboratory Medicine WA, Nedlands, Western Australia. David McGechie; PathWest Laboratory Medicine WA, Fremantle, Western Australia. Alison Keed; Royal Perth Hospital, Perth, Western Australia.

*China*: Haihui Huang; Huasahan Hospital, Fu Dan University, Shanghai. Fei Liu; Shanghai East Hospital, Shanghai. Yao-Zong Yuan; Ruijin Hospital, Shanghai Jiaotong University School of Medicine, Shanghai. Kaichun Wu; Fourth Military Medical University, Xijing Hospital, Xi’an, Xian Shi. Zhihua Ran; Shanghai Renji Hospital, Shanghai. Yunsong Yu; Sir Run Run Shaw Hospital, Zhejiang University College of Medicine, Hangzhou, Zhejiang. Jinghang Xu; Peking University First Hospital, Beijing. Ye Chen; Southern Medical University Nanfang Hospital, Guangzhou.

*Hong Kong*: Owen Tak Yin Tsang; Princess Margaret Hospital, Hong Kong. Sunny Hei Wong; Prince of Wales Hospital, Shatin. Ivan Fan Ngai Hung; Queen Mary Hospital, Pok Fu Lam.

*India*: Srinivasa Madaiah; Mysore Medical College & Research Institute, Mysore, Karnataka. Nagarjuna Yarlagadda; KIMS Hospitals, Hyderabad, Andhra Pradesh. Phillip Abraham; P. D. Hinduja National Hospital and Medical Research Centre, Mumbai, Maharashtra. Pravin Gare; Chopda Medicare & Research Centre P. Ltd, Magnum Heart Institute, Nashik.

*Indonesia*: Muhammad Hussein Gasem; Rumah Sakit Umum Pusat Dr. Kariadi, Semarang.

*Japan*: Shinya Kusachi; Toho University Medical Center, Ohashi Hospital, Meguro-ku, Tokyo. Makoto Nagashima; Toho University Medical Center, Sakura Hospital, Sakura, Chiba.

*South Korea*: Soo Jung Park; Yonsei University Severance Hospital, Seoul. Sungmin Kiem; Inje University Haeundae Paik Hospital, Busan.

*Malaysia*: Christopher KC Lee, Hospital Sungai Buloh, Sungai Buloh, Selangor. Jayaram Menon; Clinical Research Centre (CRC), Queen Elizabeth Hospital, Kota Kinabalu, Sabah. Ting Soo Chow; Hospital Pulau Pinang, Pulau Pinang.

*Philippines*: Myrna Mendoza; National Kidney and Transplant Institute, Quezon City. Randy Mercado; St. Luke's Medical Center, Quezon City. Marilyn Arguillas; Davao Doctors Hospital, Davao City. Raul Destura; The Medical City, Pasig City.

*Singapore*: David Ong Eng Hui; National University Hospital, Singapore. Ang Tiing Leong; Changi General Hospital, Singapore. Ling Khoon Lin; Singapore General Hospital, Singapore.

*Taiwan*: Yi-Hui Wu; E-Da Hospital, Kaohsiung. Po-Ren Hsueh; National Taiwan University Hospital, Taipei. Yuarn-Jang Lee; Taipei Medical University Hospital, Taipei. Jen-Hsien Wang; China Medical University Hospital, Taichung. Yao-Shen Chen; Veterans General Hospital- Kaohsiung, Kaohsiung. Wen-Chien Ko; National Cheng Kung University Hospital, Tainan.

*Thailand*: Chomsri Kositchaiwat; Ramathibodi Hospital, Bangkok, Krung Thep Maha Nakhon. Varocha Mahachai; King Chulalongkorn Memorial Hospital, Bangkok, Krung Thep Maha Nakhon. Naichaya Chamroonkul; Songklanagarind Hospital, Songkla.

*Vietnam*: Nguyen Van Kinh; National Hospital of Tropical Diseases, Ha Noi. Le Thanh Hai; National Pediatric Hospital, Ha Noi. Hoang Le Phuc; Pediatric Hospital No 1, Ho Chi Minh.
